# Silver/Snail Mucous PVA Nanofibers: Electrospun Synthesis and Antibacterial and Wound Healing Activities

**DOI:** 10.3390/membranes12050536

**Published:** 2022-05-20

**Authors:** Aalaa A. El-Attar, Hamdy B. El-Wakil, Ahmed H. Hassanin, Basant A. Bakr, Tahani M. Almutairi, Mohamed Hagar, Bassma H. Elwakil, Zakia A. Olama

**Affiliations:** 1 Microbiology and Botany Department, Faculty of Science, Alexandria University, Alexandria 21544, Egypt; alya88345@gmail.com (A.A.E.-A.); zakiaolama52@gmail.com (Z.A.O.); 2 Faculty of Applied Health Sciences Technology, Pharos University in Alexandria, Alexandria 21500, Egypt; hamdy.elwakil@pua.edu.eg; 3 Agricultural Animal Pests Department, Plant Protection Research Institute, Alexandria 21616, Egypt; 4 Centre of Smart Nanotechnology and Photonics (CSNP), SmartCI Research Centre, Alexandria University, Alexandria 21544, Egypt; ahmed.hassanin@ejust.edu.eg; 5 Materials Science & Engineering Department, School of Innovative Design Engineering, Egypt-Japan University of Science and Technology (E-JUST), Alexandria 21934, Egypt; 6 Textile Engineering Department, Faculty of Engineering, Alexandria University, Alexandria 21544, Egypt; 7 Zoology Department, Faculty of Science, Alexandria University, Alexandria 21544, Egypt; bassant.kamal@pua.edu.eg; 8 Department of Chemistry, College of Science, King Saud University, P.O. Box 2455, Riyadh 11451, Saudi Arabia; talmutari1@ksu.edu.sa; 9 Chemistry Department, Faculty of Science, Alexandria University, Alexandria 21544, Egypt; mohamedhaggar@gmail.com

**Keywords:** *Eobania vermiculata*, silver nanoparticles, electrospinning, PVA nanofibers, antibacterial activity, wound healing dressing

## Abstract

Healthcare textiles are gaining great attention in the textile industry. Electrospun nanofibers are considered the golden soldiers due to their strength, flexibility, and eco-friendly properties. The present study aimed to evaluate the potency of polyvinyl alcohol (PVA) nanofibers loaded with newly biosynthesized silver nanoparticles (Ag-NPs) as a wound healing dressing. Chocolate-band snail (*Eobania vermiculata*) mucus (which is part of the Mollusca defense system) was used as a novel reducing and stabilizing agent. Data indicated the effectiveness of *Eobania vermiculata*’s mucus in silver nanoparticle synthesis after a 24 h incubation time. The biosynthesized AgNPs-SM showed a 13.15 nm particle size, −22.5 mV ζ potential, and 0.37 PDI, which proved the stability of the synthesized nanoparticles. *Eobania vermiculata* mucus and AgNPs-SM showed potent antibacterial activity, especially against *Pseudomonas aeruginosa*. The electrospinning technique was applied in the fabrication of PVA/AgNPs-SM nanofibers, which were homogenous with a fine diameter of about 100–170 nm and showed a significantly high antimicrobial activity. In vitro and in vivo studies revealed that PVA/AgNPs-SM nanofibers were safe and efficiently enhanced the wound healing process (typical histological picture of the proliferative phase with compact and well aligned collagen fibers in the dermal tissue after 12 days) together with bacterial growth inhibition in the infected skin area.

## 1. Introduction

Mollusks are the 2nd largest animal phylum on the plant (7% of living animals are mollusks with 100–200 thousand species) [1]. Along with the mentioned biological diversity, a chemical diversity is observed because they use divergent secondary metabolites as a communication tool and as a defense system against invaders and predators [1]. The secondary metabolites of Mollusca attracted many researchers with more than 1145 natural products discovered and isolated from mollusks in the last three decades [1]. Zinconotide has been isolated from the cone shells and it is clinically tested and approved by the Food and Drugs Administration (FDA) as a treatment for severe pain, while Brentuximab vedotin is used as a treatment for lymphoma and Hodgkin’s disease [2]. 

Gastropods such as *Helix* sp. have been used for their culinary value for centuries, and the healing properties of the snail mucus for treating burns, abscesses, and other wounds have already been mentioned by Hippocrates and Pliny [1]. More recently, investigations have indicated that the extracts from *Helix pomatia* have a broncho-relaxant effect that is induced by a significant increase in prostaglandin E2 [3]. Nowadays, snail mucus is a niche product with skin regeneration properties [4]. 

Snail mucus (SM) is secreted by the pedal gland of snails with a fibrous structure. It contains biologically active compounds that perform medical functions, such as mucin (glycosylated protein), glycol acid, natural antibiotics, and glycoprotein. Snail mucus is an attractive natural substance that is increasingly used in cosmetic creams due to its emollient, moisturizing, protective, and reparative properties. Rosanto et al. [5] investigated the wound healing effect upon using different concentrations of snail mucus after the daily application of SM gel. It was concluded that topical application of 48% and 96% snail mucus gel increased the angiogenesis during the wound healing process in Wistar rat skin. 

The electrospinning method gained great attention in the production of nanofibers from polymer solutions, especially in healthcare textiles and medical field applications [6]. Polyvinyl alcohol (PVA) is widely used in biomedical applications due to its excellent biodegradability, biocompatibility, chemical resistance, and good fiber-forming properties [7]. Great attention has been directed towards hybrid polymer materials’ electrospinning with the utilization of some transition metal nanoparticles, e.g., Au, Pt, and Ag in the PVA nanofiber [8]. Among them, Ag is the most attractive metal because of its low human toxicity and strong activity against a wide spectrum of bacteria.

The aim of the present study was to newly fabricate novel wound batches of PVA/silver nanoparticles/snail mucus composites, explore the in vitro and in vivo antimicrobial activity, and evaluate the wound healing efficiency of the fabricated nanofibers.

## 2. Materials and Methods

### 2.1. Microorganisms

All the bacterial isolates used throughout the present work, namely *Acinetobacter baumanii*, *Escherichia coli*, *Enterococcus faecalis*, *Pseudomonas aeruginosa*, and *Staphylococcus aureus*, were kindly provided and phenotypically identified by the Surveillance Microbiology Department’s strain bank at the main University Hospital, Alexandria University in Alexandria, Egypt.

### 2.2. Snail Samples and Mucus Collection

Healthy snails were collected from the agricultural and garden areas of Alexandria North Coast, Egypt during the Fall of 2019. The snails were kept in wooden boxes (35 cm width × 30 cm height) with 30 snails per box. The boxes were sprinkled daily with water to maintain the humidity and the snails were on a diet of *Lactuca sativa* leaves. 

### 2.3. Snails’ Identification 

The snails were identified by the department of Zoology, Faculty of Science, Alexandria University, according to EL-Wakil et al. [9] and Godan [10].

### 2.4. Snails’ Mucus Collection 

The mucus was collected through stimulating the snails pedal glands using a sterile needle. The mucus samples were lyophilized and stored at −20 °C for further use [11]. The snails’ euthanasia was not necessary to obtain the mucus; thus, the animals were set free to the agricultural and garden areas after mucus collection.

### 2.5. Snail Mucus Composition 

A lyophilized mucus sample was hydrolyzed and purified with boiling hydrochloric acid, then the amino acid composition was determined using an amino acid analyzer (Hitachi L8900) [12].

### 2.6. Preparation of Silver Nanoparticles—Snail Mucus Nanocomposite Dispersion

Silver nanoparticles were synthesized by mixing snail mucus (10 mL) with silver nitrate solution (Merck, Darmstadt, Germany) —10 mL (25 mM). The flask was kept under shaken conditions (250 rpm) at different time intervals to assess the optimum time needed to synthesize silver–snail mucus nanoparticles. The synthesized silver nanoparticles in the snail-mucus matrix (the resultant composite form) were termed as AgNPs-SM. The formed nanoparticles were collected by centrifugation at 12,000 rpm for 20 min then washed with distilled water and lyophilized for further analyses [13]. 

#### Physico-Chemical Characterization of AgNPs-SM

The optical characterization of the prepared nanocomposite was performed by using a UV–visible spectrophotometer. The particle size (PS), polydispersity index (PDI), and Zeta potential of AgNPs-SM were determined by a dynamic light scattering (DLS) technique (Malvern Zetasizer, Worcestershire, UK) [14]. The ultra-structure, size, and shape of AgNPs-SM were examined by transmission electron microscopy (TEM) [15,16].

### 2.7. Antibacterial Activity of Snail Mucus and AgNPs-SM

The antibacterial activity of snail mucus and AgNPs-SM (25 mg each) was investigated using the disc-diffusion method [14], minimum inhibitory concentration (MIC) values, and the bacterial lethality curve [17]. 

### 2.8. Manufacturing of Nanofibrous Membranes

**Figure 1:**
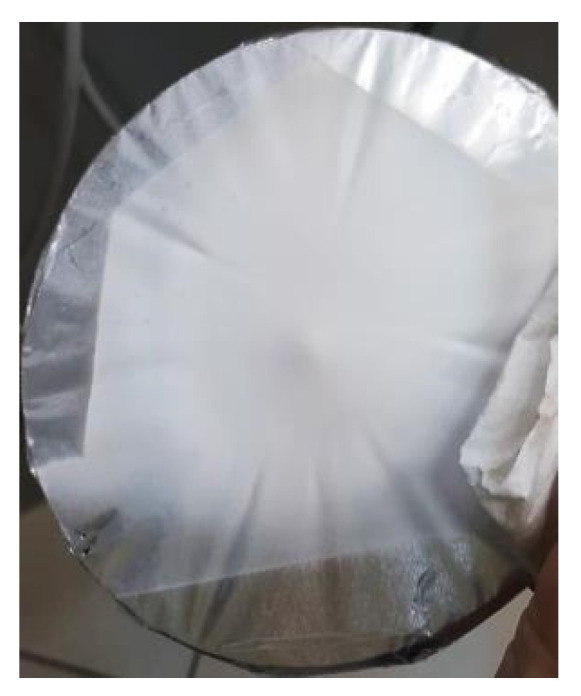
The fabricated PVA/AgNPs-SM nanofibrous dressing.

Ten grams of polyvinyl alcohol (PVA) (Merck, Darmstadt, Germany) were dissolved in 100 mL of distilled water with continuous stirring at 80 °C for an hour. Snail mucous and AgNPs-SM (3% each) were blended one at a time with the prepared PVA solution with continuous stirring (300 rpm, at room temperature for 3 h). Electrospinning of the prepared solutions were performed by using a plastic syringe with gauge 18 stainless-steel needle while a high voltage power supply (25 kV) CZE1000R (Spellman, Hauppauge, NY, USA) was applied with a flow rate of 1 mL/h [18] (Figure 1)**.**

#### 2.8.1. Morphological and Physical Characterizations of the Prepared Electrospun Nanofibers

The morphology of the electrospun nanofibers were investigated using scanning electron microscopy (SEM) (JEOL, JSM-6010LV-SEM, Tokyo, Japan). The average fiber diameter and distribution were measured using software (Madison, WI, USA) [19].

#### 2.8.2. Loading Analysis

The loading efficiency (LE%) of AgNPs on the AgNPs-SM/PVA nanofibers was measured by dissolving a sample of the prepared nanofibers with known mass in acetic acid at room temperature, and by using UV spectroscopy at a wavelength of 390 nm, the LE% was calculated according to the following equation (Equation (1)) [20]:

(1) $$\mathrm{LE}\%\;=\frac{\mathrm{Initial}\;\mathrm{drug}\;\mathrm{concentration}-\mathrm{Loaded}\;\mathrm{drug}\;\mathrm{concentration}}{\mathrm{Initial}\;\mathrm{drug}\;\mathrm{concentration}}\times 100.\;$$

### 2.9. In Vitro Studies 

#### 2.9.1. Release Profile Assay

The release profile of AgNPs from PVA/AgNPs-SM nanofibers was measured. The PVA/AgNPs-SM nanofiber samples were cut into 2 × 2 cm pieces and incubated in phosphate buffer saline solution (PBS, pH 7.0) under shaking at 50 rpm and 37 °C. AgNPs release was measured at specific time intervals (15 min, 30 min, 1, 2, 4, 6, 12, 24, 48, and 78 h) at 390 nm, and the drug concentration was calculated and expressed as a cumulative percent of the released AgNPs [20].

#### 2.9.2. Evaluation of the Cytotoxic Effect of the Prepared Electrospun Nanofibers

The human normal melanocyte cell line (HFB4 cells) was obtained from the American Type Culture Collection (ATCC, Rockville, MD, USA). The cells were grown on RPMI-1640 medium supplemented with 10% inactivated fetal calf serum and 50 µg/mL gentamycin. The cells were maintained at 37 °C in a humidified atmosphere with 5% CO_2_ and were subcultured two to three times a week. For cytotoxicity assays, the cell lines were suspended at a concentration of 5 × 10^4^ cell/well in Corning® 96-well tissue culture plates, and then incubated for 24 h. The tested compounds were then added to 96-well plates (three replicates) to achieve eight concentrations of each tested substance. After incubating for 24 h, the numbers of viable cells were determined by the MTT test. The relation between surviving cells and drug concentration was plotted to get the survival curve of each cell line after treatment with the specified substance. The cytotoxic concentration (CC_50_), i.e., the concentration required to cause toxic effects in 50% of the intact cells, was estimated from graphic plots of the dose response curve for each tested concentration using Graphpad Prism 5 software (San Diego, CA, USA) [21,22].

#### 2.9.3. Antibacterial Activity of the Prepared Electrospun Nanofibers

The antibacterial activity was investigated according to ASTME 2149-01 (Standard Test Method for Determining the Antimicrobial Activity of Immobilized Antimicrobial Agents under Dynamic Contact Conditions). The antimicrobial activity of the synthesized nanofibers was expressed as the reduction percent of each test organism after 24 h incubation with the nanofibers under test according to the following formula (Equation (2)):

(2) $$R\;\left(\%\right)=\frac{B-A}{B}\times 100.\;$$

R% is the reduction percentage of the microbial colony number, A is the number of microbial colonies in the flask containing the nanofibers after 24 h incubation, and B is the initial number of the microbial colonies in the flask prior to the nanofibers addition [22]. Moreover, the bacterial lethality curve was assessed by placing 1 cm × 1 cm of the tested nanofibers inside bacterial suspension tubes (10^2^ CFU/mL), then 100 µL of the bacterial suspension was plated on agar plates at different incubation time intervals and, after 24 h of incubation, the number of formed colonies were counted [23]. Moreover, PVA/AgNPs-SM nanofiber antibacterial activity was assessed by a scanning electron microscope (SEM). PVA/AgNPs-SM nanofiber samples with standard dimensions (1 cm × 1 cm) were added to *Pseudomonas aeruginosa* suspension (10^2^ CFU/mL). After 24 h of incubation, the nanofibers were collected and examined under an electron microscope [24]. 

### 2.10. In Vivo Skin Wound Healing Experiment 

#### 2.10.1. Animals and Ethics

Fifty male rats (*Rattus norvegicus albinus*) were purchased from the Experimental Animal House of Pharos University—Alexandria, Egypt. Male rats weighing 180–220 g were recruited. Rats were split into five experimental groups (10 rats/each). The rats were kept at the same temperature and humidity all the time with a 12 h light–dark cycle and were acclimatized to the environment for at least a week before the experiment. All the procedures for the care and handling of the animals were approved by the Animal Care and Use Committee at the Faculty of Science (AU/06/20/04/25/9/02), Alexandria University, Alexandria, Egypt.

#### 2.10.2. Wound Healing Assessment

The wound healing ability of AgNPs-SM was evaluated using full thickness skin defects in *Pseudomonas aeruginosa* skin infected rats (intradermal injection of 20 μL 7.27 × 10^9^ CFU/mL 48 h prior to wounding). The tested rats were anesthetized with isoflurane and then full-thickness excisional skin wounds were done with a sterile 10 mm biopsy punch. The rat groups were treated daily for 7 days with 1 cm × 1 cm AgNPs-SM/PVA, SM/PVA, and PVA nanofibers individually (applied directly to the wound site as a wound dressing to cover the wound in the treated group and changed daily). The wound size on day 0 was set to 100%, and each subsequent day was reported as a percentage of the initial wound size. At day 4, 8, and 12 post-wound, the rats were scarified for histological analysis. The recovery percentage (Equation (3)), epidermal structure, collagen bundles, dermal organization, and epidermal regeneration were observed [23].

(3) $$\mathrm{Recovery}\;\mathrm{percentage}\;=\frac{\mathrm{Wound}\;\mathrm{surface}\;\mathrm{area}\;\mathrm{on}\;\mathrm{day}\;X}{\mathrm{Wound}\;\mathrm{surface}\;\mathrm{area}\;\mathrm{on}\;\mathrm{day}\;0}\times 100$$

#### 2.10.3. Bacterial Load Assessment

At specific time intervals (days 4, 8, and 12 post-infection), rats were sacrificed. The tissue samples were weighed and homogenized in 1 mL of tryptic soy broth (TSB). Each tissue homogenate was serially diluted in polyphosphate buffer (PPB) then plated on blood agar plates. The plates were then incubated at 37 °C for 24 h, viable *Pseudomonas aeruginosa* cells (CFU/g tissue) were counted, and the bacterial reduction in the skin wound post-treatment was determined for each group according to the following equation (Equation (4)) [23,24].

(4) CFU/g=Platecount (1|dilution)×10/Tissue weight

#### 2.10.4. Histologic Examination

The rats from all treatment groups were euthanized by excessive isoflurane inhalation on day 4, 8, and 12 after wounding. The wound and the surrounding intact skin were harvested, and each sample was bisected at the center of the wound. The tissue samples were fixed in 10% formalin, embedded in paraffin, and sectioned into 3 μm thick slices. At least six serial sections near the center of the wound were obtained per wound. Sections for histologic examination were deparaffinized, rehydrated, and stained with hematoxylin & eosin (H&E) and Massons Trichrome individually [20].

### 2.11. Statistical Analyses

All data were analyzed using SPSS 17.0 software (IBM, NY, USA) and expressed as the mean ± SD. One-way analysis of variance (ANOVA) and student Newman–Kells tests were used to analyze the differences among groups. At *p* < 0.05, the difference was deemed to be statistically significant. All graphs were drawn by Origin 9.1 software (Origin Lab., Northampto, MA, USA).

## 3. Results and Discussion

### 3.1. Composition of Snail Mucus

The amino acid contents of *Eobania vermiculata* mucus proved to contain high concentrations of glutamic and aspartic acid (3.36 and 3.35 mg/gm, respectively) (Table 1). The composition of snail mucus usually varies according to the differences in the snail species and the mucus function (adhesion or protection). Typically, the snail mucus consists of 90–99.7% water. The snail mucus ideally contains glycosaminoglycans, hyaluronic acid, glycoprotein enzymes, and antimicrobial peptides. Alanine and leucine are well-known for their desquamating capabilities as well as their key function to enhance cellular proliferation and wound healing, while glycine has an outstanding ability to penetrate skin and promote collagen formation [25]. 

### 3.2. Preparation and Characterization of Silver Nanoparticles—Snail Mucus Nanocomposite Dispersion

**Figure 2:**
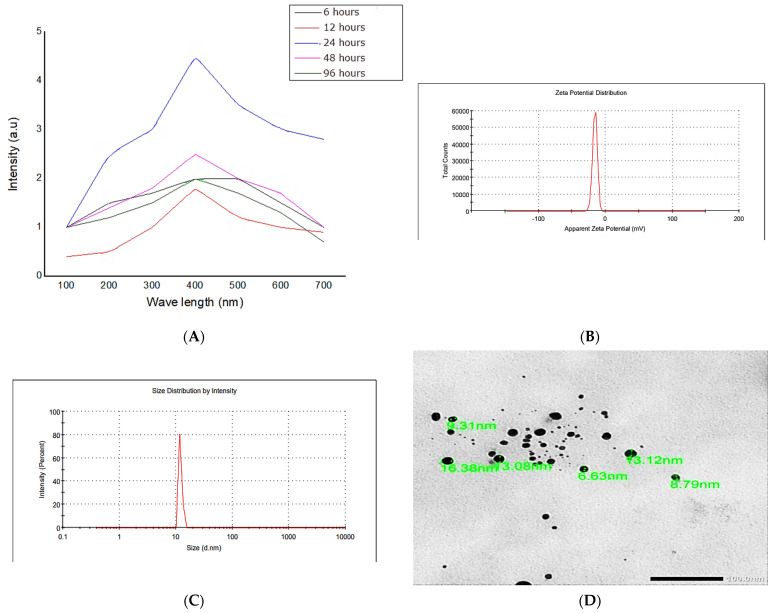
UV-Visible absorbance (**A**), Zeta potential (**B**), Zeta size distribution (**C**), and TEM micrograph (**D**) of the biosynthesized AgNPs using the snails’ mucus extracts.

Silver nanoparticles were newly biosynthesized by *Eobania vermiculata* mucus. The preparation process was monitored at different time intervals to assess the optimum reaction time. Data revealed that the optimum reaction time was 24 h of incubation between the 25 mM silver nitrate solution and 10 mL snail mucus (Figure 2). Therefore, the reaction time was fixed to 24 h in further analyses. The biosynthesized AgNPs-SM had a 13.15 nm particle size, −22.5 mV ζ potential, and 0.37 PDI, which proved the stability and the relative homogeneity of the synthesized nanoparticles. 

Gubitosa et al. [26] examined the capacity of snail slime from garden snails “*Helix aspersa* Müller” to synthesis biomedical gold nanoparticles (AuNPs-SS), which had an average particle size of 14.6 nm. The safety of the synthesized AuNPs-SS on human keratinocytes, as well as their potential influence on wound healing and anti-inflammatory characteristics in murine macrophages, was reported with increased keratinocyte adhesion, spreading, and migration, while LPS-induced cytokine levels (IL1- and IL-6) decreased and the generation of inducible nitric oxide synthase (iNOS) was entirely inhibited.

### 3.3. Antibacterial Activity of Snail Mucus and AgNPs-SM

*Eobania vermiculata* mucus and AgNPs-SM showed potent antibacterial activity against the tested strains. The highest antibacterial activity of the biosynthesized AgNPs-SM was observed against *Pseudomonas aeruginosa* (65 mm inhibition zone diameter and 2 µg/mL minimum inhibitory concentration) (Table 2). The *Pseudomonas aeruginosa* strain was selected for further analyses.

The mucus of *Eobania vermiculata* was reported to have an effective antibacterial effect against two different strains of *Pseudomonas aeruginosa* as well as *Streptococcus pyogenes* [27]. *Eobania vermiculata* mucus inhibited the growth of *Escherichia coli*, *Staphylococcus aureus*, and *Staphylococcus epidermidis*, but no activity was noticed against *Pseudomonas aeruginosa* and *Enterococcus faecium* [25]. Ag nanoparticles’ antibacterial activity may be elucidated by the Ag ions’ entry into bacterial cells via diffusion and osmosis, causing cell enzyme and protease denaturation, followed by bacterial cell destruction [19,28].

### 3.4. Fabrication and Characterization of Nanofibrous AgNPs-SM/PVA

**Figure 3:**
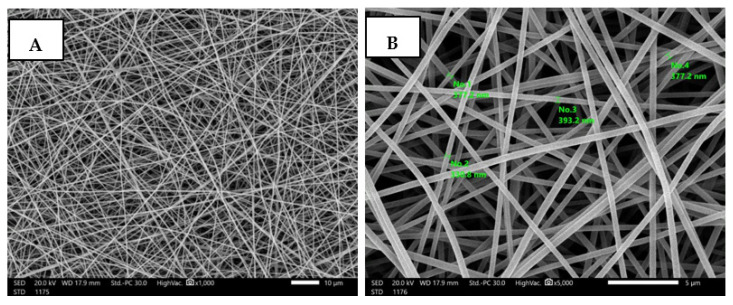

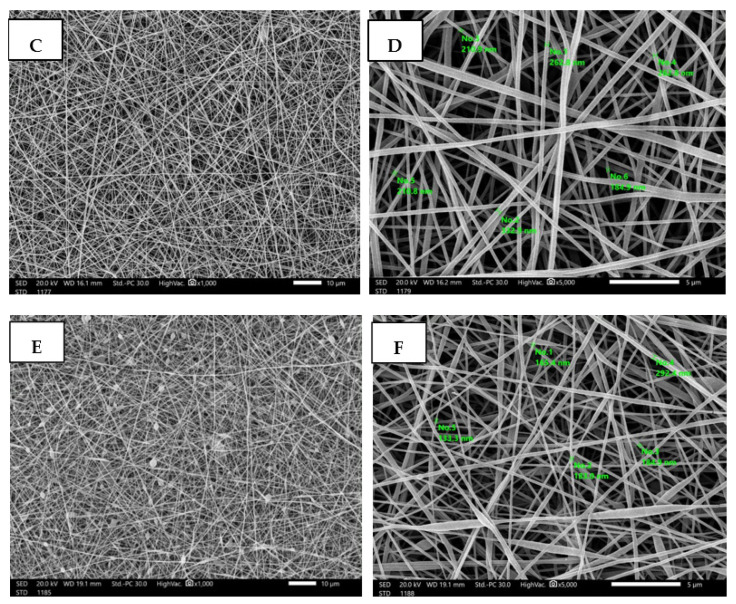
SEM images of PVA (**A**,**B**), PVA/SM (**C**,**D**), and PVA/AgNPs-SM nanofibers (**E**,**F**).

The PVA composite nanofibrous membrane morphology was examined by scanning electron microscope (SEM). The average fiber diameter of the nanofibers was obtained using Image-J software. The SEM images revealed that the AgNPs-SM/PVA nanofibrous membrane had a homogenous structure with a fine diameter of about 100–170 nm. The addition of nanoparticles did not affect the fiber diameter as a result of the good dispersal, small particle size, and optimized spinning conditions (Figure 3, Table 3). 

According to Leung et al. [29], the increased solution viscosity generated by SM addition is connected to an increase in the average diameter, and the ideal SM concentration was 2% (*w*/*v*), which significantly lowered the nanofiber average size diameter and strengthened the membrane homogeneity. According to Kim et al. [30] the insertion of bioactive molecules inside the nanofiber matrix may be efficiently regulated by utilizing the electrospinning technique. 

#### 3.4.1. Fourier Transform Infrared Spectroscopy (FT-IR) Characterization

**Figure 4:**
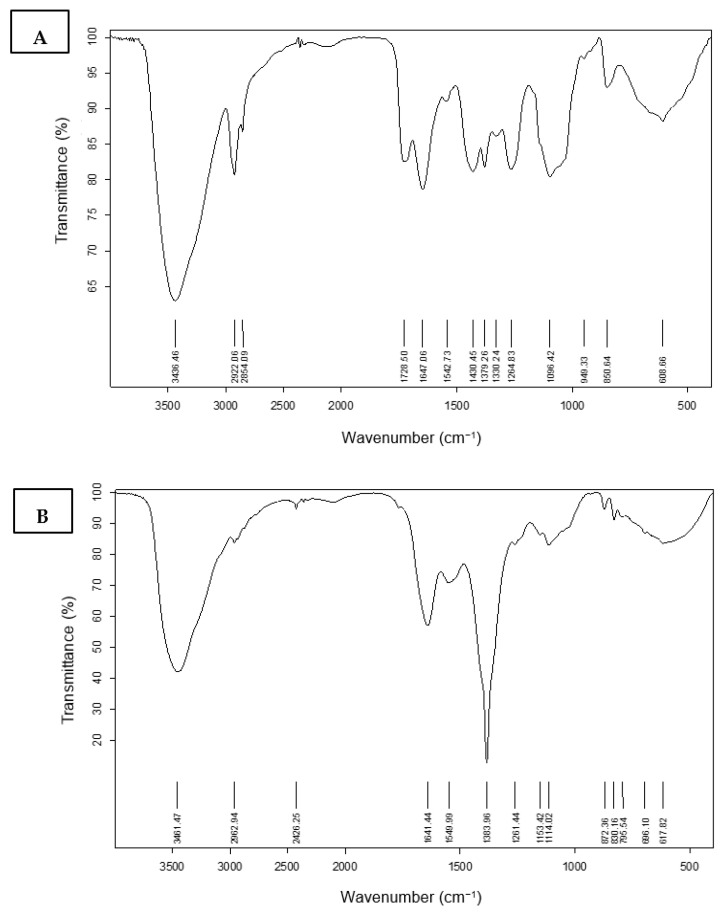

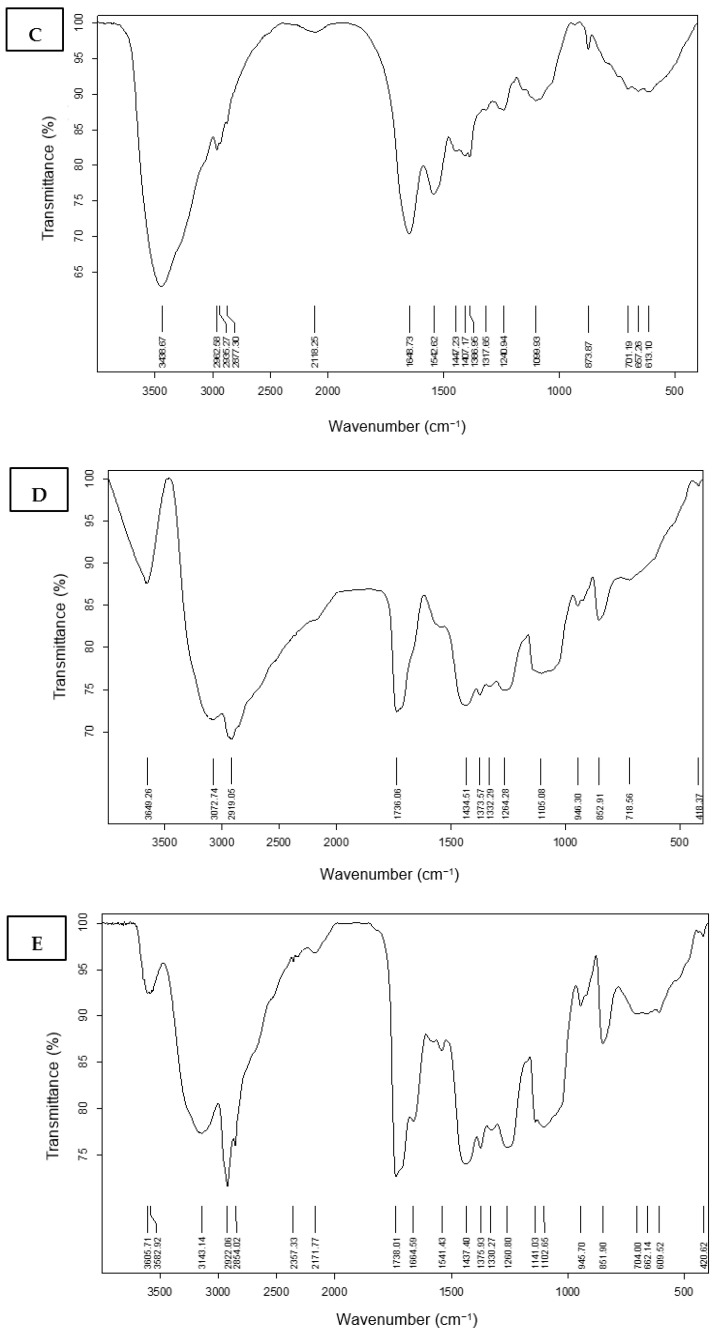
FT-IR of (**A**) PVA, (**B**) Ag nitrate, (**C**) snail mucus, (**D**) PVA/AgNPs-SM, (**E**) PVA/SM.

The infrared spectra for the region of 700–4000 cm^−1^ of prepared nanofibers of PVA, Ag nanoparticles, and snail protein with and without combinations are shown in Figure 4. Additionally, as shown in Figure 4a, PVA has characteristic peaks at 715, 849, 1096, 1430, 1647, 2922, and 3436 cm^−1^. The IR peaks at 715 and 871 cm^−1^, respectively, are caused by out-of-plane vibrations of the O–H and C–H bonds. The IR peaks at 1096 and 1430 cm^−1^ are caused by C–O stretching and bending vibrations of the CH_2_ group, respectively. An IR peak at 1728 cm^−1^ is due to the C=O stretching vibrations of the residual non-hydrolyzed vinyl acetate group of the PVA. An IR peak at 2922 cm^−1^ is due to the stretching vibration of the CH_2_ group. The strong peak at 3436 cm^−1^ is caused by the OH stretching vibrations of strong hydrogen bonds from intramolecular and intermolecular forms. IR spectra of silver nanoparticles, on the other hand, revealed a strong band of the OH stretching band at 3461 cm^−1^, as well as two additional shoulder peaks at 1641 cm^−1^ and 1383 cm^−1^ (Figure 4b). The presence of a very strong band at 3438 cm^−1^ in Figure 4c corresponds to the snail protein’s N-H stretching vibration, whereas the amidic carbonyl group stretching vibration was present at 1648 cm^−1^. The silver-embedded PVA fibers were accompanied by a considerable increment in the vibrational frequency allocated to in-plane O–H vibrations, with C–H vibrations at 3649 and 2922 cm^−1^. This figure depicts how implanted silver interacts with the PVA fiber matrix O–H group. Figure 4d showed an out-of-plane O–H vibration with a large peak at a lower wave number of 852 cm^−1^. On the other hand, as shown in Figure 4e, the presence of the snail mucus in the nanofiber of the PVA showed a significant effect on the position and the shape of the vibrational peaks of either the PVA or the mucus. The stretching vibration of the N-H and O-H groups is shown at 3605 and 3583 cm^−1^. Another effect was also shown at the C=O stretching vibration at 1738 cm^−1^, with 10 wave number increments due to the presence of the proteinic combination. Additionally, the presence of the protein in the PVA matrix affords an increment of the amidic C=O group of the snail protein to 1664 cm^−1^. The higher wave number of these bonds could be explained in terms of the intermolecular interaction of the N–H and O-H with C=O groups of either the PVA or the mucus. 

#### 3.4.2. Loading Analysis and Release Profile

**Figure 5:**
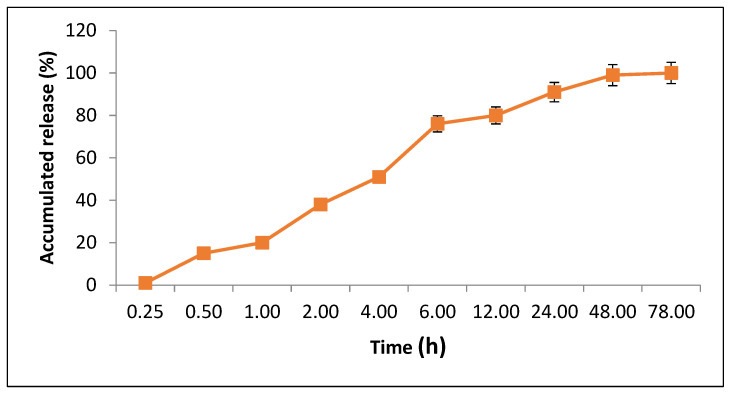
AgNPs release behavior from the AgNPs-SM/PVA nanofiber.

The silver nanoparticle loading efficiency of the prepared PVA/AgNPs-SM was 69.7%. On the other hand, the release behavior of AgNPs from the prepared PVA nanofibers was evaluated in PBS for 78 h. A sharp release was observed during the first 6 h with a sustained release that lasted until 78 h (Figure 5). 

### 3.5. Evaluation of Cytotoxic Effects of the Synthesized AgNPs-SM 

**Figure 6:**
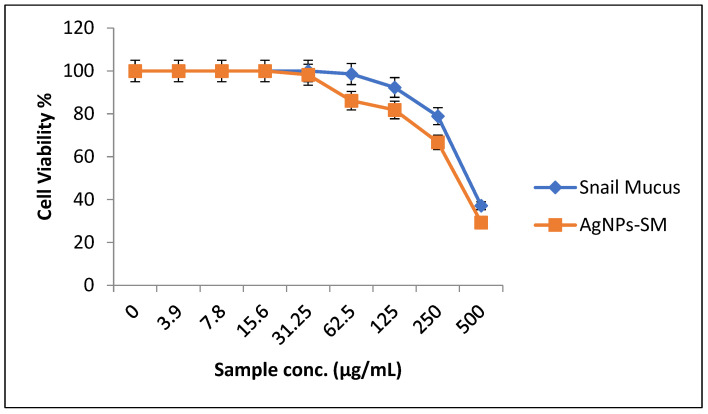
The percentage of normal melanocytes cell viability as affected by snail mucus and AgNPs-SM.

In a trial to study the in vitro cytotoxic effect of the biosynthesized AgNPs-SM, cell proliferation using the human normal melanocyte cell line (HFB4 cells) was investigated. It was found that at 500 µg/mL of snail mucus, the melanocyte cell viability was 37.14% and the CC_50_ was 423.08 µg/mL. While at 500 µg/mL of AgNPs-SM, the cells viability was 29.3% and the AgNPs-SM CC_50_ was 390.42 µg/mL (Figure 6). 

The in vitro cell proliferation and migration of *Helix aspersa* snail mucus using a human keratinocyte cell line (HaCaT cells) and primary dermal fibroblasts (HF) were assessed by Iglesias-de la Cruz [31]. Snail mucus enhanced the HaCaT cell proliferation and migration in a time- and dose-dependent manner. Furthermore, snail mucus therapy promoted migratory behavior, adhesion molecule expression, and increased the cell viability by promoting FAK phosphorylation and -catenin nuclear localization in both HaCaT and HF. 

### 3.6. Antibacterial Activity of the Prepared Nanofibers

**Figure 7:**
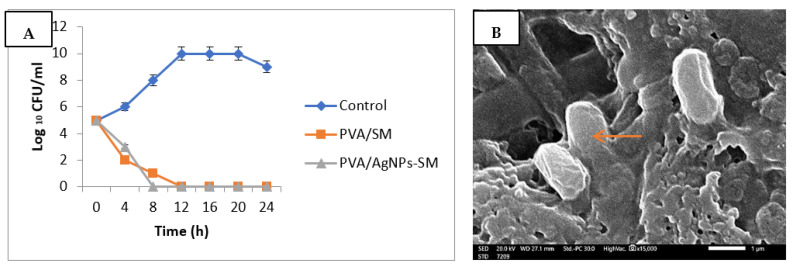
Antibacterial activity of PVA/AgNPs-SM nanofibers: Bacterial lethality curve (**A**), Scanning electron microscopic (SEM) image (**B**) (Arrow showing lysed bacterial cell).

*Pseudomonas aeruginosa* was the most susceptible strain and was chosen for further analyses. The antibacterial activities of the prepared nanofibers were assessed through the ASTME 2149-01 Standardized technique. The growth reduction% of *Pseudomonas aeruginosa* after 24 h of incubation with the PVA/AgNPs-SM nanofibers was 85.4%, with the ability to inhibit bacterial growth after 8 h (Figure 7A). The SEM study was used to determine the mechanism of action of PVA/AgNPs-SM nanofibers. The results revealed that *Pseudomonas aeruginosa* cells were adsorbed and adhered to the PVA/AgNPs-SM nanofiber surface, which led to bacterial cell lysis and the release of the cell’s contents (Figure 7B).

The observed antibacterial effect may be attributed to the relatively small average diameter size of the prepared nanofibers that allowed the bacterial absorption through the hydrogen bonding force and static electricity, which resulted in blocking bacterial growth and led to microbial cell deaths [28].

### 3.7. In Vivo Experiments

#### 3.7.1. Skin Wound Healing Experiment 

**Figure 8:**
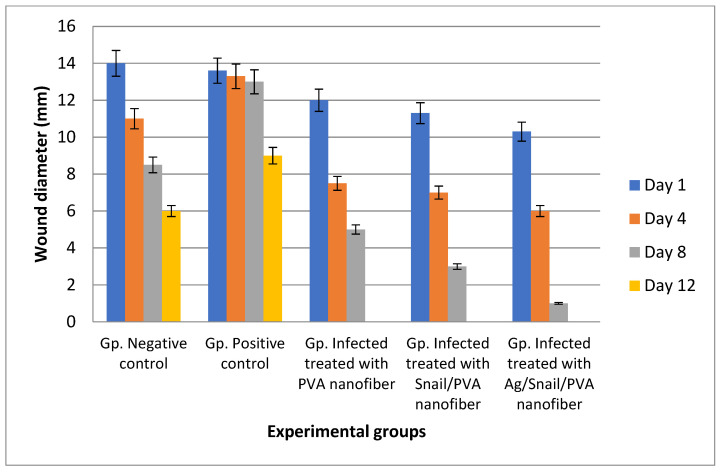
Wound healing area as affected by time intervals.

Wound contraction in infected rats showed a gradual reduction of the wound area in the PVA/AgNPs-SM nanofibers compared to the control groups. Day 12 post-injury under treatment with PVA/AgNPs-SM nanofibers revealed 100% contraction of the wound area (Figure 8, Table 4).

#### 3.7.2. Bacterial Load Assessment

**Figure 9:**
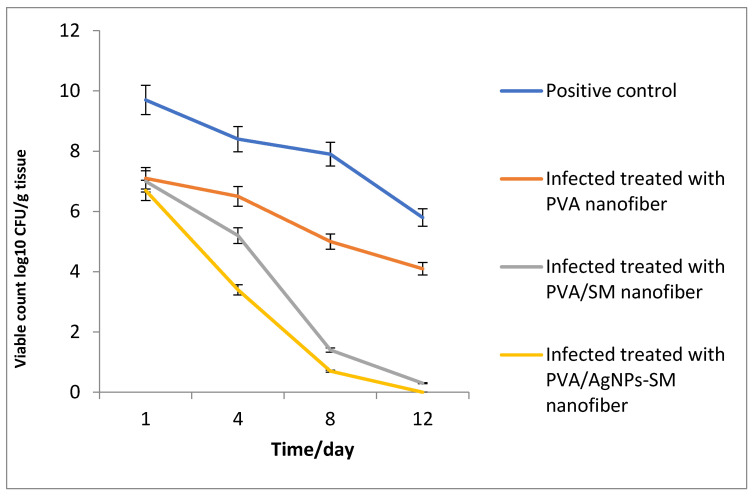
Bacterial cell load for the treated *P. aeruginosa* wound infections.

At the beginning of the experiment, the rats received an intradermal injection containing 7.27 × 10^9^ CFU/mL of *Pseudomonas aeruginosa*. Furthermore, at a specific time interval, the viable *Pseudomonas aeruginosa* cells were counted (CFU/g tissue) and the bacterial reduction in the infected skin wound post-treatment was determined for each group. Data in Figure 9 showed a significant reduction in the bacterial cell count among all the treatments in relation to the control groups.

#### 3.7.3. Histological Study

##### Normal Skin Histology

**Figure 10:**
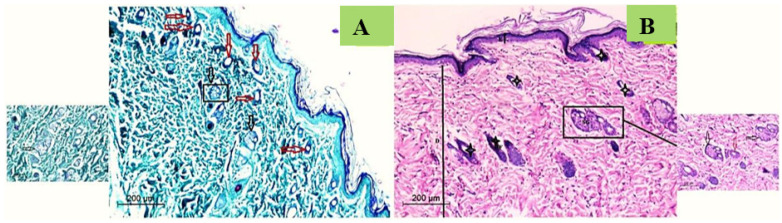
Photomicrograph of histological appearance of normal rat skin stained with Massons Trichrome (**A**) and H&E (**B**), Magnification 200×. Transparent black arrows refer to sebaceous gland; transparent red arrows refer to sweat gland; strikes refer to mature hair follicles.

Typical skin rats displayed a normal thin ordered epidermal layer. Hair follicle clusters (vellus and guard hairs) and accompanying sebaceous glands were abundant. Additionally, there was a consistent pattern of adipose connective tissue with the underlying muscle fibers. Massons Trichrome stain revealed densely packed, regularly orientated, thick bundles of collagen in the dermal layer (Figure 10).

##### Wound Degree

**Figure 11:**
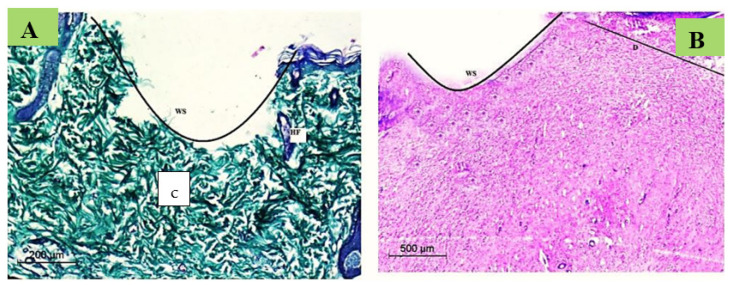
Photomicrograph of the histological appearance of immediate skin wounded stained with H&E (**A**) and Massons Trichrome (**B**). In which: D (Dermis); WS (wound site); c (collagen); HF (hair follicle).

Histopathological sections were investigated to confirm the wound degree in the current study immediately after the wound induction. It was revealed that the wound degree was severe with no epidermis, yet the damage spread to the early dermis layers, thereby illustrating the extent of this wounding method’s devastation (Figure 11).

##### Histological Observation of Skin of the Negative Control Wounded Rats (Group 1)

**Figure 12:**
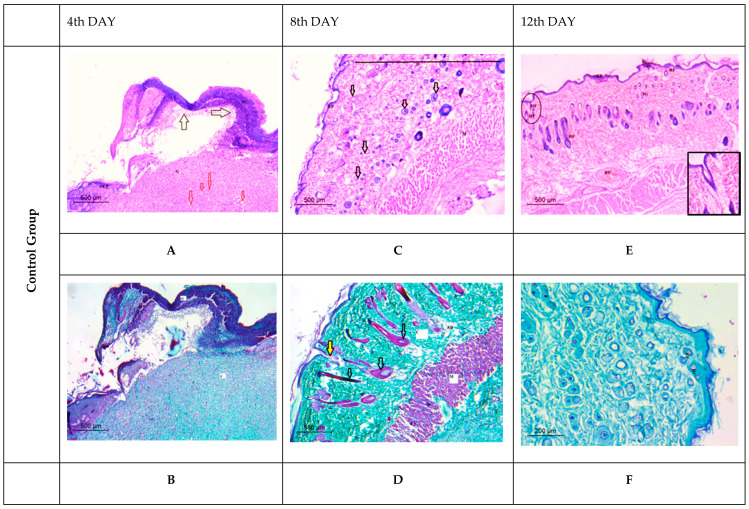

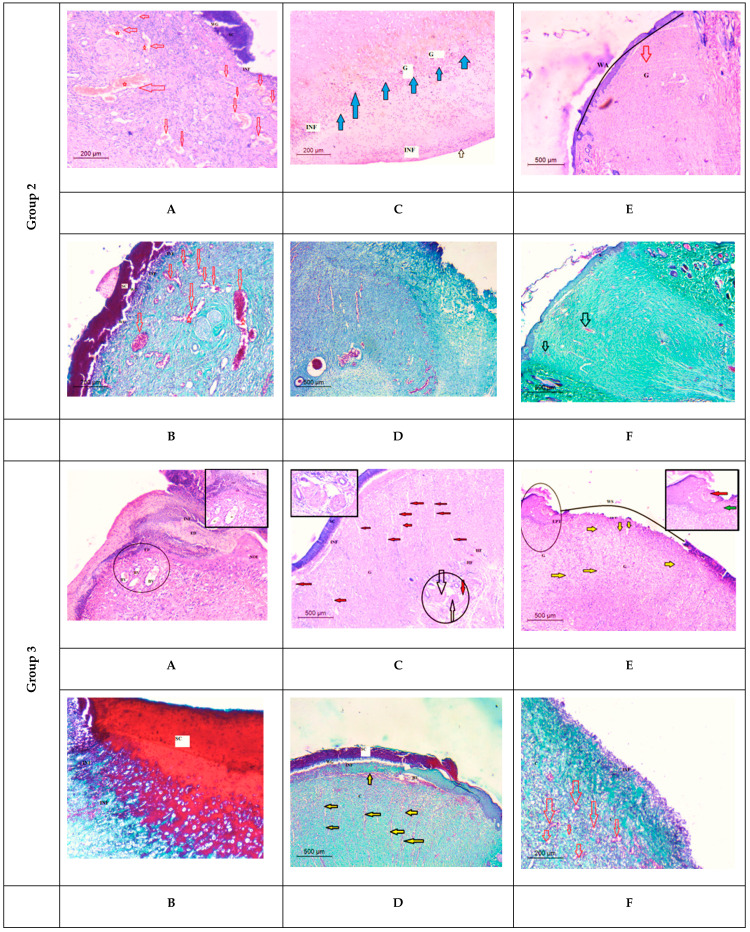

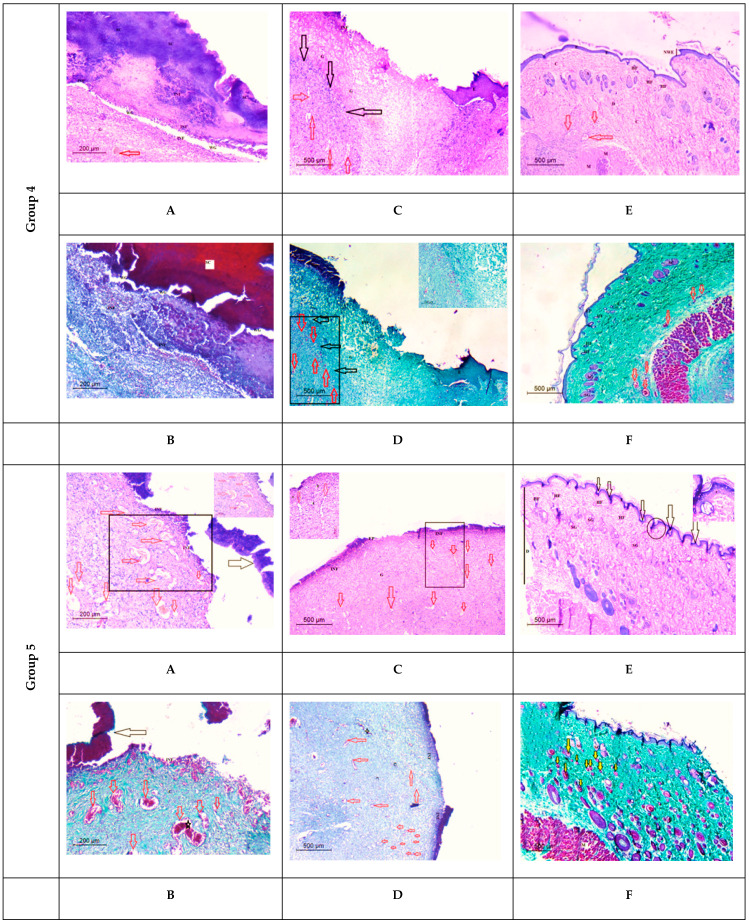
Histological appearance of different wounded rat groups stained with H&E (**A**,**C**,**E**) and Massons Trichrome (**B**,**D**,**F**) at different time intervals (4th, 8th, and 12th post-wound), in which: SC (Scab); BV (Blood vessel); INF (Inflammatory cells); OE (original epidermis); WS (wound site); G (granulation tissue) yellow arrows (hair follicles); red arrows (blood vessels); ED (Edema); WA (wound area), EPT (epidermal tongue); HF (hair follicle); arrows refer to blood vessel (angiogenesis); C (collagen); stars reefers to dilated blood vessels. Note: Group 2:arrows refer to blood vessel (angiogenesis); (**C**): blue arrows refers to boundaries between provisional and mature granulation tissue. Group 3: arrows refer to blood vessel (angiogenesis); (**C**,**D**): transparent arrows refer boundaries between the provisional and mature granulation tissue. Group 4: (**A**,**B**): transparent arrows refer to detached scab, (**E**) transparent arrow refers disorganized epidermal layer. Group 5: (**C**): transparent arrows refer to sebaceous gland, (**E**): red arrow refers to scab detached; green arrow referred to epidermal tongues.

The negative control group (non-infected and non-treated rats) revealed significant damage to the epidermis and dermis tissue at the 4th day post-wound induction. A big scab encompassed the wounded spot and there was a conspicuous dilated blood vessel (Figure 12A). The dermis showed virtually little collagen deposition when stained with Masson’s Trichrome (Figure 12B). Early granulation tissue production and the emergence of several hair follicles were seen near the wound gap’s base (Figure 12C). A substantial degree of collagen deposition was visible using Masson’s Trichrome stain (Figure 12D). The last interval findings demonstrated that a re-growth of the epidermal layer was accomplished to a significant extent on the 12th day after wounding. Furthermore, both the dermal tissue and the sebaceous glands had a typical morphology, and there was a regular pattern of adipose connective tissue and collagen deposition (Figure 12E,F). 

##### Histological Observation of Skin of the Positive Control Wounded Rats (Group 2)

The first interval sections revealed partial scab detachment; the inflammatory process was more severe and widely spread in the wound area, with a large number of inflammatory cells present and extensive necrosis observed (Figure 12A). The dermal layer revealed less significant collagen deposition with angiogenesis prevalence for subsequent healing stage advancement using Masson’s Trichrome stain (Figure 12B). On the 8th day post-wound, granulation tissue was seen in the dermal layer, with a visible demarcation between the provisional and mature granulation tissue matrix (Figure 12C,D). In the last interval, epidermal growth failed to achieve re-epithelialization. The basement membrane was poorly defined or missing in different locations, thereby resulting in a disorganized basal epidermis. The severe inflammatory infiltration in the dermis had reduced significantly and was accompanied by necrotic alterations and loss of all skin appendages, which indicated impaired wound healing in comparison to the control and treated groups (Figure 12E,F). 

##### Histological Observation of Skin of the PVA Nanofiber-Treated Wounded Rats (Group 3)

The healing pattern showed the appearance of a large scab attached directly to the dermis remnants on the 4th day after wounding. Inflammatory cells were also detected as diffused at the wound site with wound exudate exacerbating edema, which was a prominent feature in this group (Figure 12A). Using Masson’s Trichrome stain (Figure 12B), the dermal papillary layer revealed a fine, loosely arranged network of collagen fibers. On the 8th day after wound induction, the skin had no discernible epithelium (chaotic epidermal cells) and the dermal–epidermal junction was irregular, with an abundance of small blood vessels (angiogenesis) allowing cellular proliferation and granulating tissue generation (Figure 12C). A dilated blood vessel underlying the epidermis was recognizable using Masson’s Trichrome stain (Figure 12D). Furthermore, on the 12th day after the wound, there were no signs of re-epithelization and epidermal eshar was detached from the dermis, leaving remnants and edema at the wound edges (Figure 12E). Newly developed granulation tissue was distinguished by relative intense collagen deposition (many and numerous mature aspect fibers), as observed by the Masson’s Trichrome stain (Figure 12F).

##### Histological Observation of Skin of the PVA/SM Nanofibers Treated Wounded Rats (Group 4)

On the 4th day post wounding, a large scab area was seen completely detached over the disorganized dermal tissue. The epidermis was entirely destroyed and disrupted. A considerable number of dilated blood vessels and some granulation tissue were also present, indicating an early wound healing process (Figure 12A,B). On the 8th day post wounding, inflammatory cells were noticed, and the number of dilated blood vessels was reduced, which also signifies the onset of the overlapped remodeling phase (Figure 12C). Clear deposition of collagen fibers in the dermal layer was apparent using Masson’s Trichrome stain, along with dilated blood vessels (Figure 12D). On the 12th day post wounding, many well-formed sebaceous glands were seen in the dermal layer, along with hair follicles that were located between muscle tissue and adipose tissue, thereby demonstrating the healing stage (Figure 12E). By using Masson’s Trichrome stain, collagen fibers were more organized and deposited in the dermal layer surrounded by hair follicles and glands (Figure 12F)

##### Histological Observation of Skin of the PVA/AgNPs-SM Nanofiber-Treated Wounded Rats (Group 5)

On the 4th day post wounding, the healing pattern showed that the large scab was attached directly to the dermis remnant. Furthermore, inflammatory cells were observed at the wound site, whereas the appearance of a narrow-wound gap emphasized the scab’s close detachment. The dermal layer showed a limited presence of blood vessels (Figure 12A). Masson’s Trichrome stain confirmed the inflammation at the wound site as a healing stage. The dermal layer, in particular, revealed an unorganized collagen pattern of tattered dermal remains (Figure 12B). On the 8th day post wounding, a large scab was detached from the newly progressed epidermal layer, explored the thickened edges of epidermis, and keratinocytes migrated beneath the detached scab (epidermal tongue). An observable granulation tissue confirmed the wound’s early remodeling. The boundary between the provisional matrix and the newly formed matrix was clearly observed (Figure 12C). In the upper layer of the dermis, sections revealed an irregular arrangement of collagen. As well, an intense proliferation and dense collagen deposition were noticed by using Massson’s Trichrome stain (Figure 12D). The last interval findings revealed a significant degree of re-epithelization, showing the epidermal organization and thickness (comparable to the intact epidermis). The epidermal–dermis tissue boundary was clearly visible with the maturation of numerous hair follicles (Figure 12E). This interval showed a typical histological picture of the proliferative phase with compact and well-aligned collagen fibers in the dermal tissue (Figure 12F).

Snail mucus possesses cell development capabilities through its promoting and regenerating properties, which have been utilized in medicine to treat burn injuries, pain alleviation, and other ailments. Snail mucus has an unusual combination of natural ingredients (allantoin and glycolic acid) with skin-healing qualities. Previous studies reported that snail mucus demonstrated, as compared to gelatin/chitosan PVA nanofibers, improved water absorption capacity and flexibility, as well as superior and regulated degradability. As a result, snail mucus might be employed in tissue engineering [32].

Statistical evidence gave some data about the potential use of mucus in wound treatment. *Eobania vermiculata* mucus includes antioxidant superoxide dismutase (SOD) and Glutathione-S-Transferase activity (GST). Antioxidants are compounds that may protect cells from harm (produced by the free radicals or reactive oxygen species). SODs operate as antioxidants, preventing reactive oxygen species from oxidizing cellular components. Moreover, the snail mucus prompted the proliferation of fibroblasts, extracellular matrix assembly, and regulated the metalloproteinase activities. Further research found that the snail mucus boosted the cell–cell and cell–substrate adhesion molecules’ motility and expression in mammalian fibroblast and keratinocyte cells [30]. It should be emphasized that some of these qualities are similar to those claimed for several current wound care solutions [33]. 

Ganesh et al. [34] prepared PVA-chitosan composite electrospun nanofibers that were co-encapsulated with Ag nanoparticles and sulfanilamide for enhanced wound-healing effect. The scanning electron microscopy (SEM) images of the nanofibers revealed an average fiber diameter of 150 nm, which was suitable for the encapsulation of Ag nanoparticles as a treatment of microbial infected wounds. The in vivo wound healing evaluation using a rat model revealed that wound closure reached 90.76 ± 4.3% after 7 days. Alipour et al. [35] fabricated nanofibers of PVA-pectin loaded with Ag nanoparticles and showed high antibacterial effects against the *Escherichia coli, Pseudomonas aeruginosa*, and *Staphylococcus aureus* strains. Moreover, the in vitro cytotoxicity evaluation showed significantly high cell viability of HSF-PI 18 fibroblast cells, confirming the nanofibers’ safety. 

## 4. Conclusions

This is the first work to investigate *Eobania vermiculata* mucus in silver nanoparticle biosynthesis. Snail mucus was applied in the synthesis of novel nanofiber dressings using electrospinning (PVA/AgNPs-SM). It was revealed that PVA/AgNPs-SM nanofiber dressing efficiently enhanced the wound healing process together with bacterial growth inhibition in the infected skin area. This may pave the way to synthesize metal nanoparticles and formulate biodegradable polymeric electrospun wound dressings using snail mucus.

## Figures and Tables

**Table 1:** Amino acid contents of *Eobania vermiculata* mucus.

Amino Acid	Concentration (mg/gm)
Aspartic acid	3.350
Therionine	1.376
Serine	1.489
Glutamic acid	3.360
Proline	1.754
Glysine	1.241
Alanine	1.522
Valine	1.167
Methionine	0.051
Iso leucine	0.835
Leucine	2.303
Tyrosine	1.655
Phenyl alanine	2.044
Histidine	2.565
Lysine	1.053

**Table 2:** Antibacterial activity of the snail mucus and the biosynthesized AgNPs-SM.

Tested Strains	Inhibition Zone Diameter (mm)		MIC (µg/mL)	
	SM	AgNPs-SM	SM	AgNPs-SM
*Enterococcus faecalis*	45.0	60.0	128.0	4.0
*Pseudomonas aeruginosa*	49.0	65.0	64.0	2.0
*Acinetobacter baumanii*	44.0	50.0	128.0	32.0
*Escherichia coli*	37.0	52.0	128.0	16.0
*Staphylococcus aureus*	48.0	58.0	64.0	16.0

**Table 3:** Average Fiber Diameter (nm) of the electrospun nanofibers.

Nanofiber	Average Fiber Diameter (nm)
PVA	170
PVA/SM	126
PVA/AgNPs-SM	110

**Table 4:** Morphological characterization of wound area as affected by time.

Tested Groups	Day 1	Day 4	Day 8	Day 12
Negative control	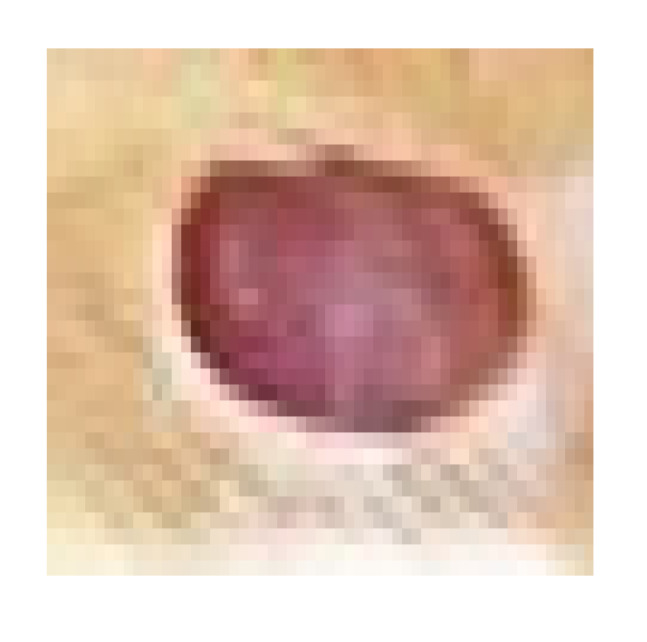	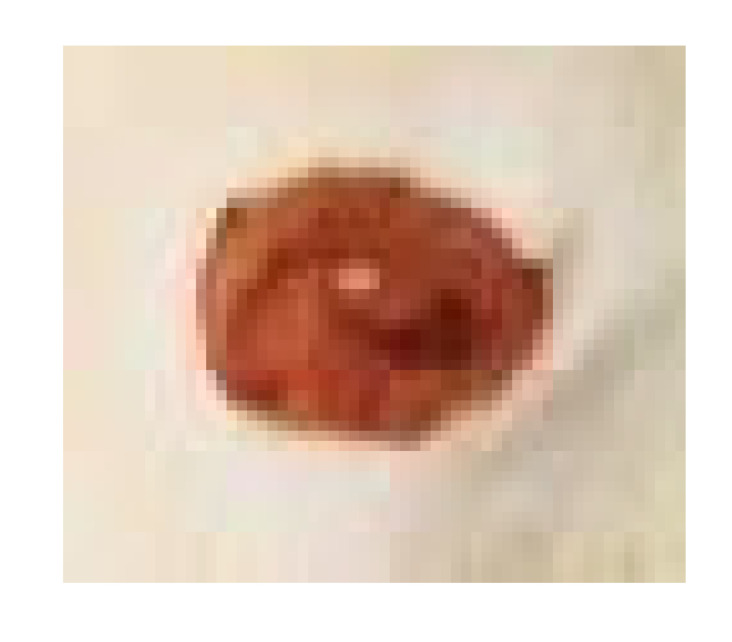	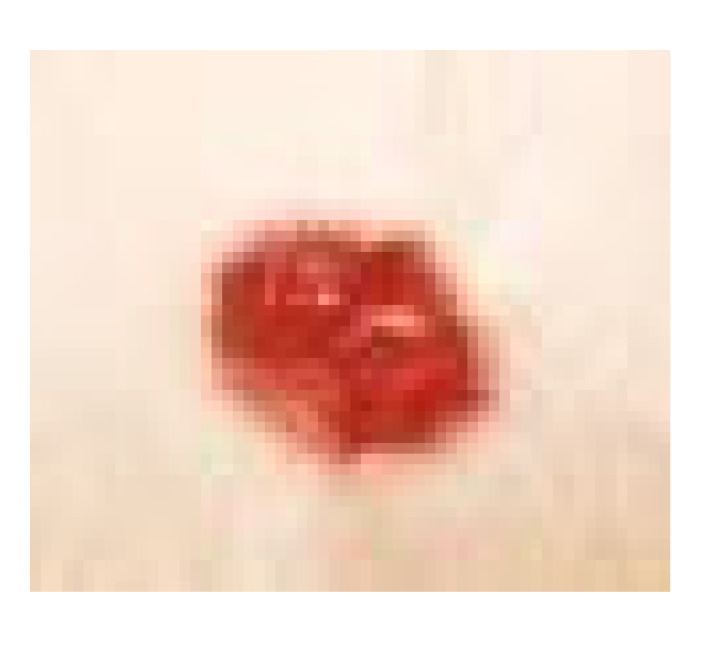	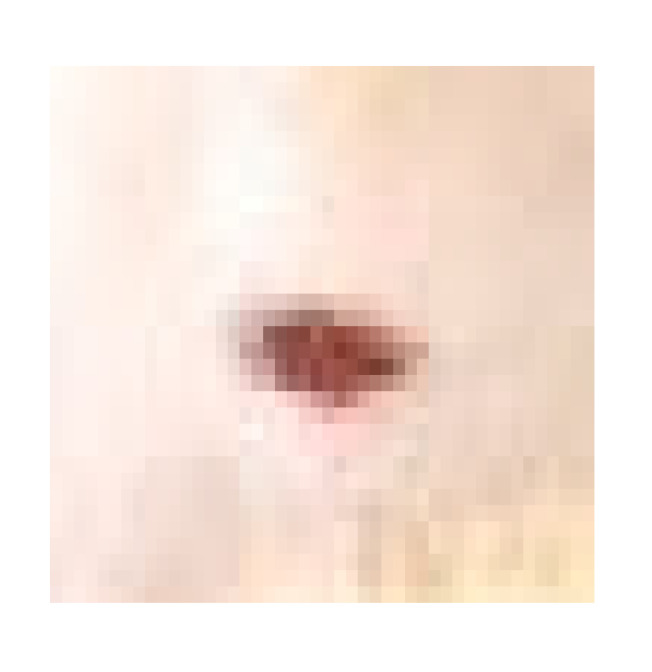
Positive control		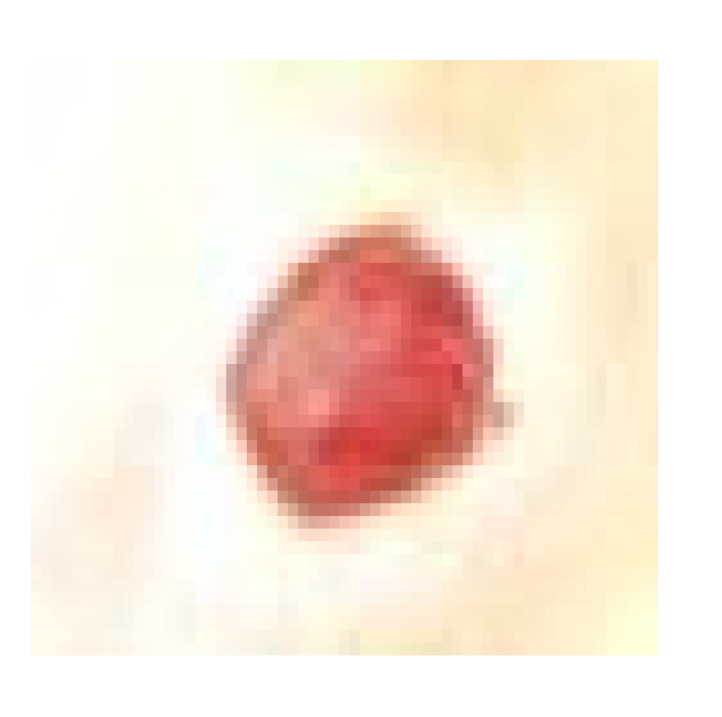	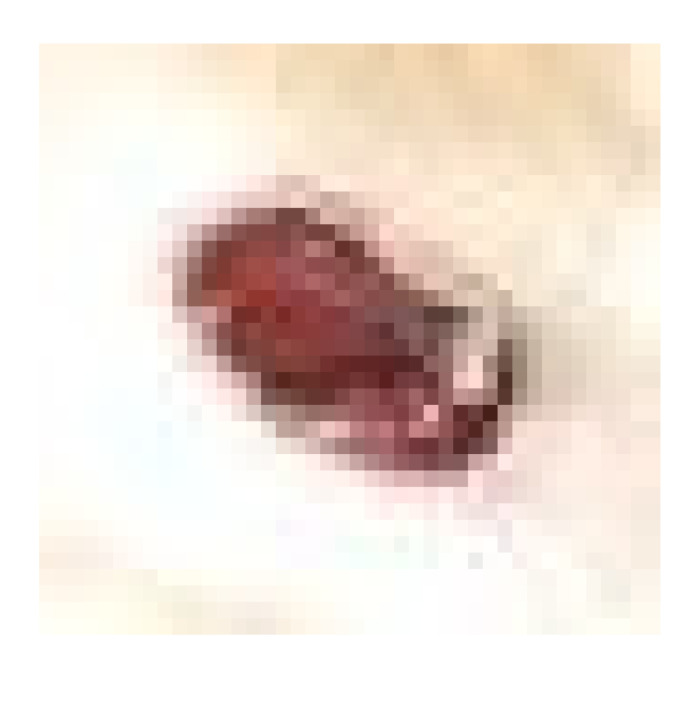	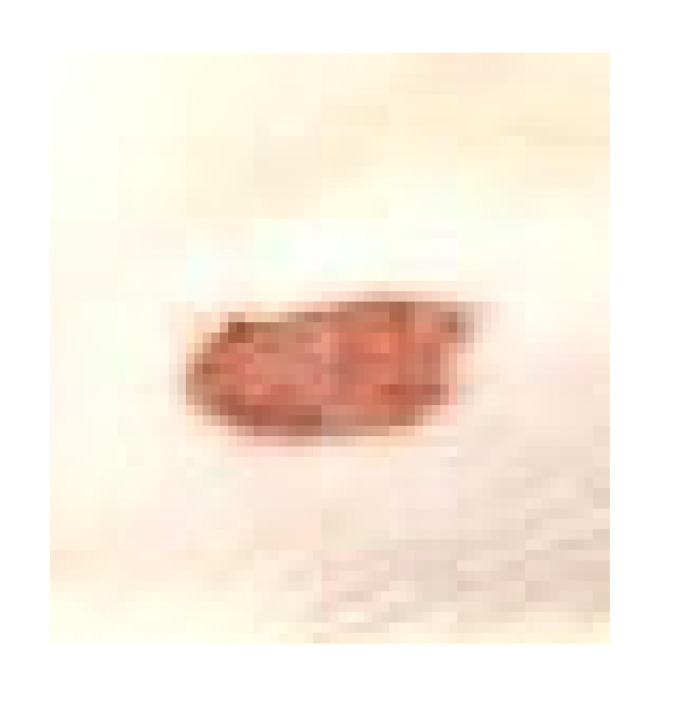
Infected + treated with PVA nanofiber		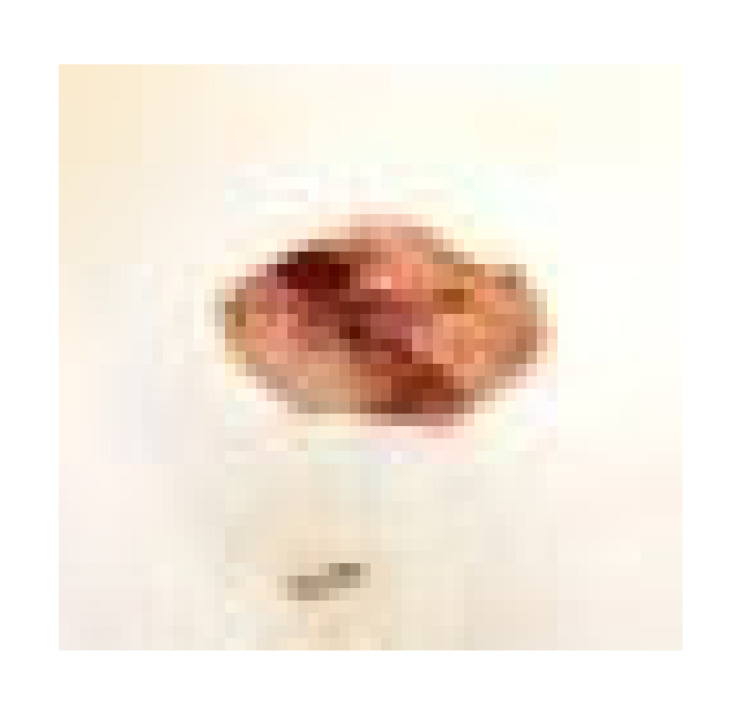	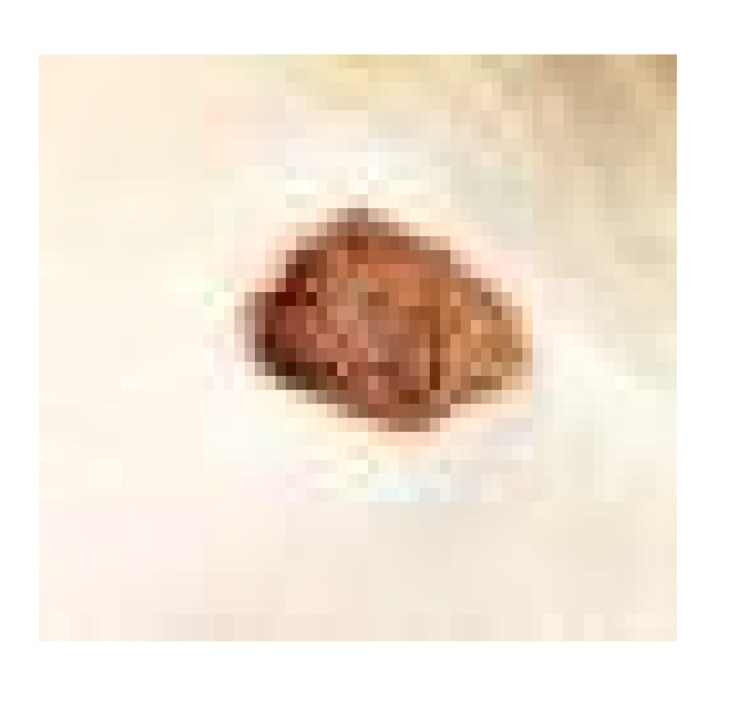	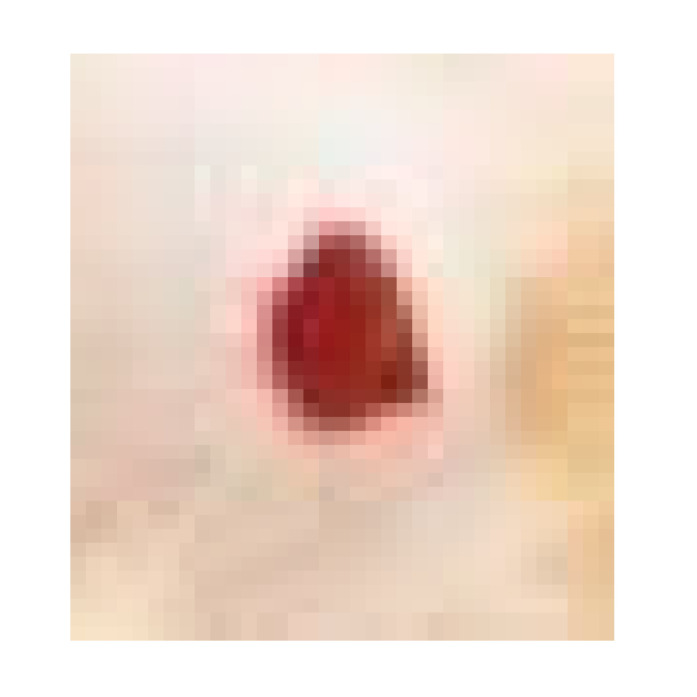
Infected + treated with PVA/SM nanofiber		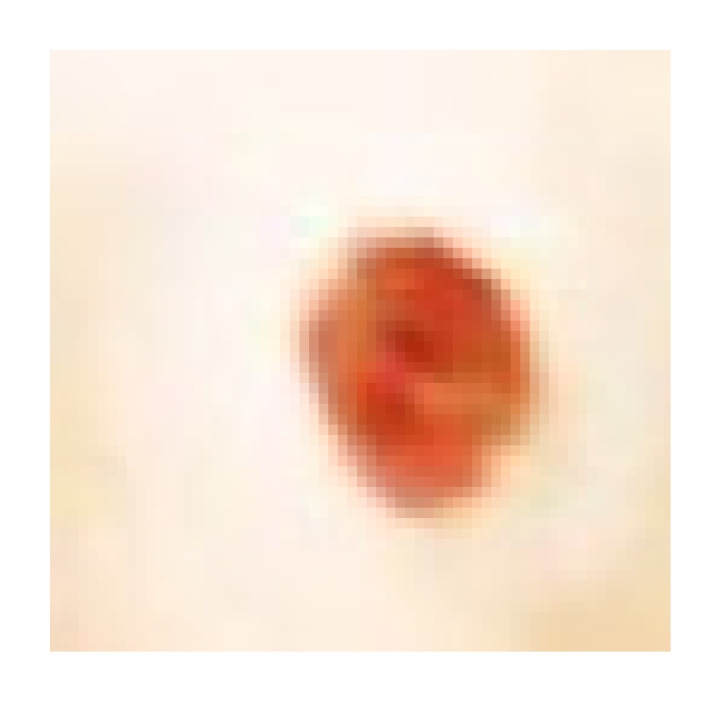	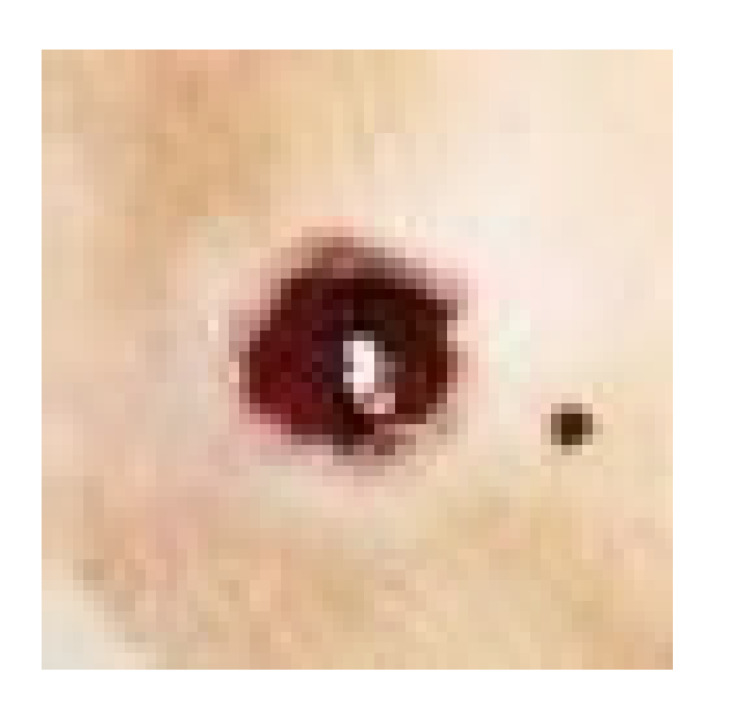	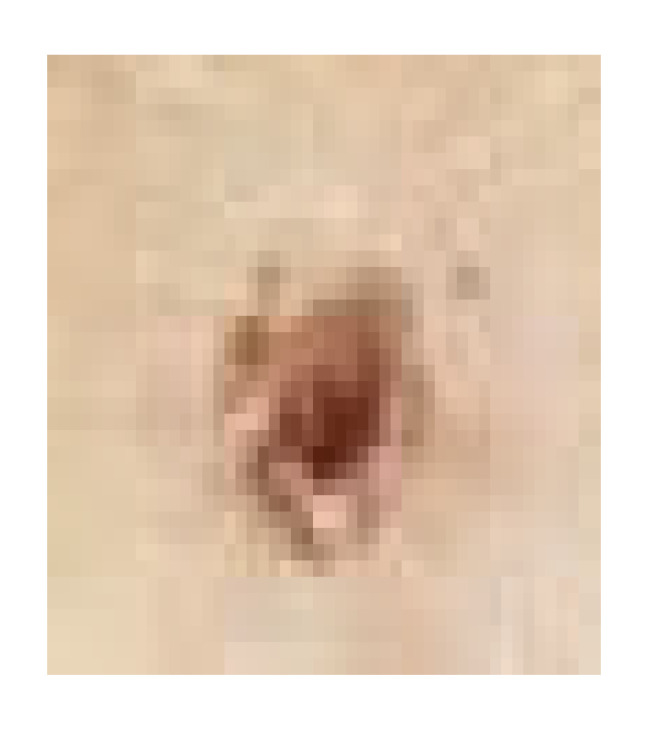
Infected + treated with PVA/AgNPs-SM nanofiber		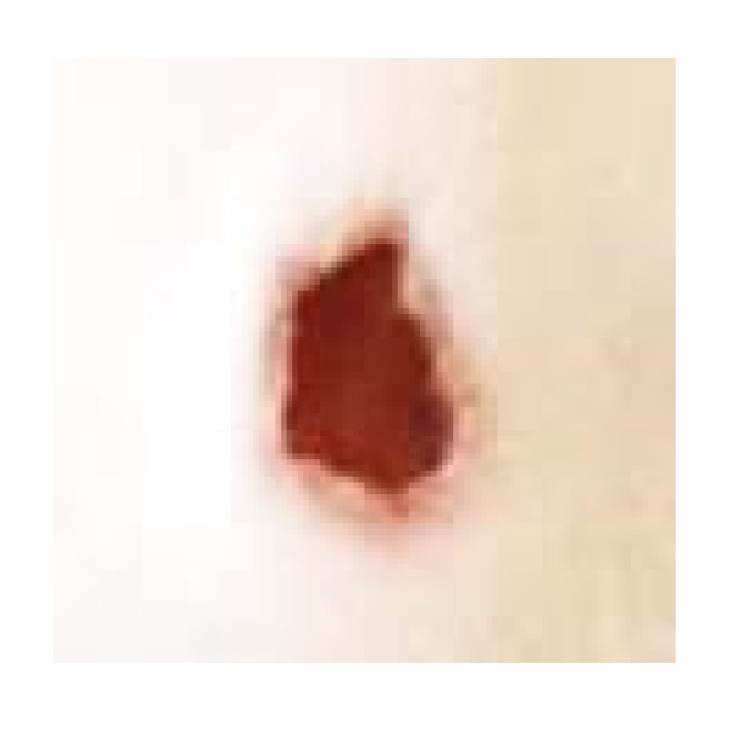	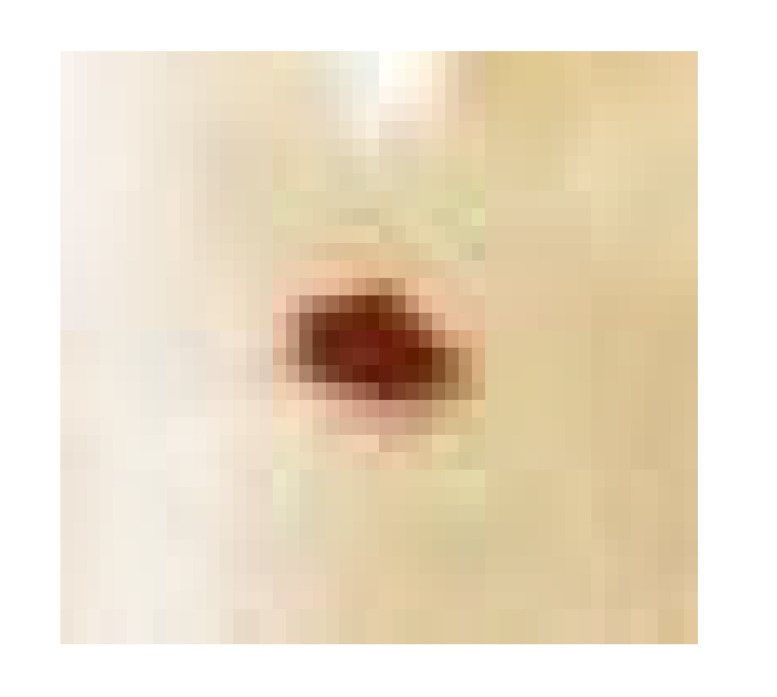	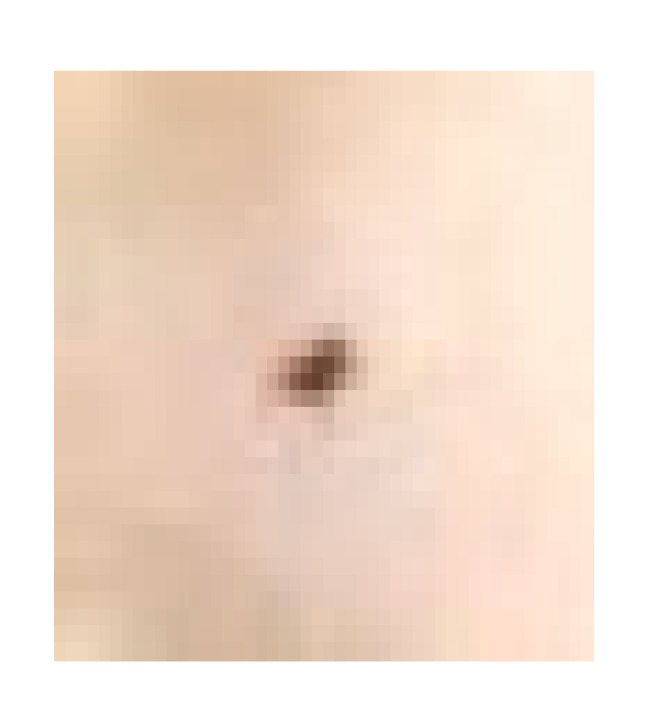

## Data Availability

Not applicable.
